# Angular-Dependent THz Modulator with Hybrid Metal-Graphene Metastructures

**DOI:** 10.3390/nano13131914

**Published:** 2023-06-23

**Authors:** Huan Wang, Jiajun Linghu, Xuezhi Wang, Qiyi Zhao, Hao Shen

**Affiliations:** 1 School of Science, Chang’an University, Xi’an 710061, China; linghujiajun@chd.edu.cn (J.L.); xzh_wang@chd.edu.cn (X.W.); 2 School of Science, Xi’an University of Posts & Telecommunications, Xi’an 710121, China; qiyi_xiyouphy@163.com

**Keywords:** graphene, metastructure, terahertz, modulation

## Abstract

The coupling effects of surface plasmon resonance (SPR) from metamaterials induce variation in both the frequency and intensity of plasmonic modes. Here, we report an angular-dependent THz modulator with hybrid metal–graphene metastructures. The metastructures composed of the period gold split-rod arrays on top of a monolayer graphene, which show redshift modulation in the THz region with an increasing incident angle due to the strong out-of-plane magnetic flux introduced by the clockwise circular current at the oblique incidence. By utilizing graphene-based actively tunable conductor with ion-gel electrical gating, the THz transmission can be significantly modified. The modulation depth of the hybrid metal–graphene metastructure modulator can reach ~37.6% at 0.62 THz with a gate voltage of −3 V. The theoretical modeling of transmitted dependency on frequency and incident angle is demonstrated at different Fermi energies, which fits well with the experimental results. This hybrid device can offer a useful method for THz applications (such as angle sensors or angular-resolved spectroscopy), where angle-dependent modulation is needed.

## 1. Introduction

The terahertz (THz) spectral region, serving as a bridge between electronics and optics, has attracted tremendous interest over the past few decades for its potential applications in noninvasive imaging [1], high-speed communications [2], and biology [3], etc. Benefiting from the enormous progress in THz wave generation [4] and detection [5], a surging demand for other active THz wave devices has emerged. THz modulators with high efficiency [6,7] and fast speed [8,9] are considered as one of the vital components for various applications.

Graphene, a two-dimensional structure arranged in a hexagonal lattice with sp^2^ hybridized carbon atoms, possesses exceptional gapless electronic band structure [10], high mobility [11] and linear energy dispersion [12] for electronic and optoelectronic applications [13,14]. The absorption properties of graphene in the visible to near-infrared region are dominated by the optical conductivity originating from the interband transition, which yield a constant absorption of 2.3% [15]. In the far-infrared and THz spectral region, however, the frequency-dependent optical response of graphene is governed by intraband transition [16], which can be changed by exploiting its tunable Fermi energy (*E_f_*) and well described by the Drude model [17]. Such unique characteristics make graphene a promising material for THz modulators through electrical gating. However, graphene has weak absorption due to the atomic thickness, which constrains the modulation depth. Therefore, it is desirable to enhance the modulation strength in graphene by coupling with a strongly localized field.

Metamaterials composed of discrete sub-wavelength structures have attracted enormous attention for versatile applications, such as perfect lensing [18], invisible cloaking [19], perfect absorbers [20], and sensing [21]. These artificial materials can fully control the properties of electromagnetic (EM) waves in terms of amplitude [22], phase [23], polarization [24], and frequency [25], via their underlying structures [26,27]. Consequently, research on the design and fabrication of metamaterials has been extensively focused on a wide EM spectrum from the visible to THz spectral region. Meanwhile, metastructures with broken symmetry are shown to exhibit narrow line width resonances yielding high-quality factor, which can be designed by varying the parameters [28] and modifying the coupling strength [29]. As metamaterials can exhibit a strong enhancement of near-fields, it is achievable to modulate transmitted EM wave by employing graphene as an efficiently active control to enhance localized field [30,31]. It is reported that with the electrostatic doping method, the modulation depth of hybrid graphene-metastructure device can be enhanced several times, which has been executed with an applied voltage of less than 3 V [32]. By integrating passive metamaterials with graphene, Balci et al. demonstrated a new class of electrically controlled active metadevices working in broadband frequencies [33]. However, angle-resolved hybrid graphene metastructures have remained elusive, which can be engaged to acquire many significant physical peculiarities, such as spatial dispersion [34], electromagnetically induced transparency (EIT) [35], and chirality [36]. More importantly, the symmetry of incident electromagnetic field distribution can be initiatively broken at oblique incidence, which leads to asymmetric surface current [37] and thus contributes additional coupling in metamaterials [38]. Therefore, it is necessary to conceive angular resolved spectroscopy of metastructures by using graphene to implement the active modulation.

Here, we demonstrate an angular-dependent modulator with hybrid metal–graphene metastructures in the THz region. By applying ion-gel doping, the transmission characteristics of the gated hybrid metal–graphene metastructures can be tuned, which consequently induces modulation of the transmitted wave. We observe that the resonance increases with the incident angle due to the enhanced localized field in the split-rod arrays. The THz modulation enhancement of the hybrid device is verified by both experimental and theoretical results, which shows dependency on the incidence angles and gating voltages. It turns out that the maximum modulation depth can reach −37.6% with a gate voltage of −3 V at an incident angle of 30°. Our results show that the hybrid metal–graphene metastructures can be useful in various active devices for angle-dependent photonic applications.

## 2. Materials and Methods

### 2.1. Device Design and Fabrication

**Figure 1:**
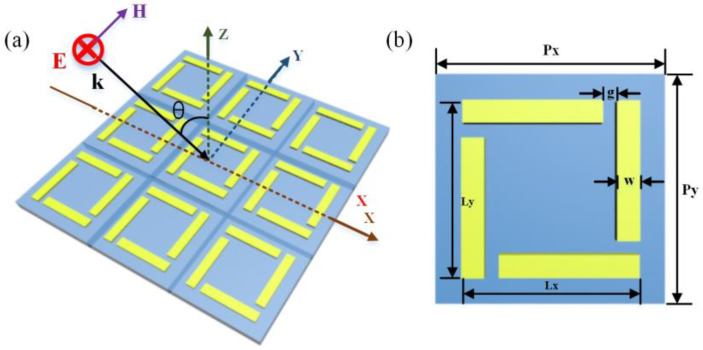
(**a**) Schematic of gold split-rod metamaterials consisting of square gold split-rod arrays at oblique incidence under polarized wave along the x direction. (**b**) The geometric parameters of the proposed metastructures are shown as follows: *P_x_ = P_y_* = 120 µm, *L_x_ = L_y_* = 100 µm, *g* = 6 um, and *w* = 10 um.

The configuration of the metamaterials is sketched in Figure 1a, which consists of square gold split-rod arrays. Due to the metastructures displaying the four-fold symmetry, the optical response under linear polarized excitation along the X- and Y-axes will be coincident. Hence, we only discuss the optical properties of the device with linear polarized incidence along Y-axis (TE mode). The geometric parameters are shown in Figure 1b, and the metal metastructures of a gold split-rod array with period (*P_x_* and *P_y_*) of 120 µm, length (*L*) of 100 µm, split width (*w*) of 10 µm and split gap (*g*) of 6 µm are designed to generate the optical resonance at range of 0–1 THz, which is to fit with the operation wavelength of our THz measurement setup. 

**Figure 2:**
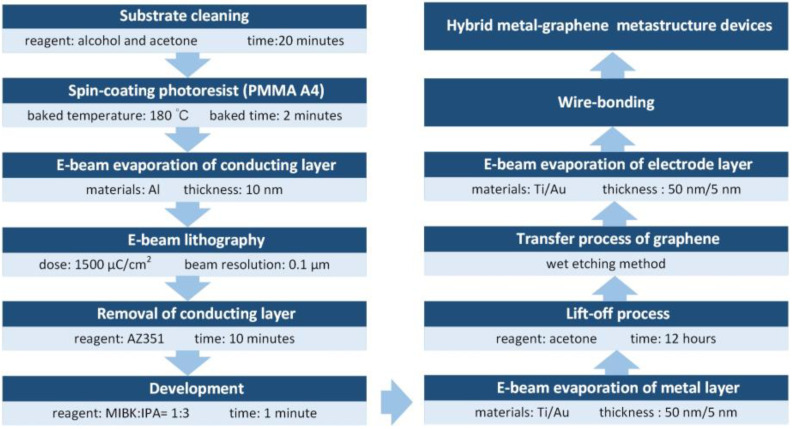
Fabrication diagram of hybrid metal–graphene metastructure.

**Figure 3:**
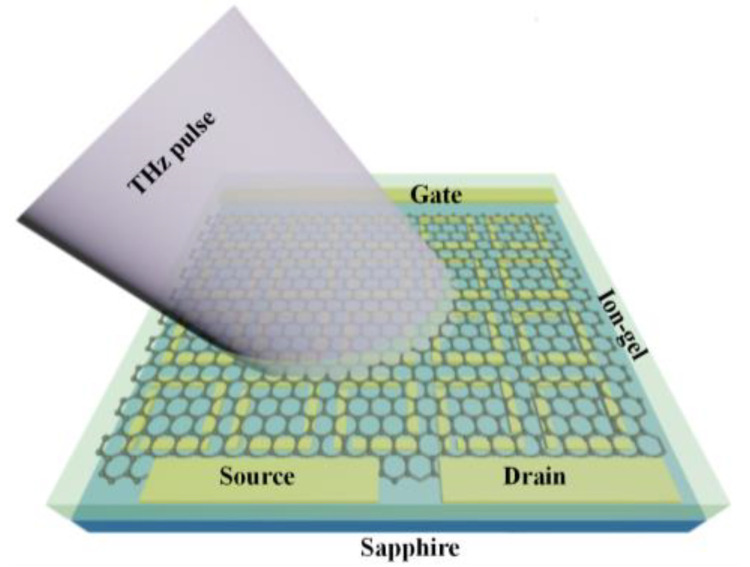
Schematic of hybrid metal–graphene metastructure device.

Experimentally, the gold split-rod metamaterials are fabricated on a sapphire substrate with further covered with chemical-vapor-deposition (CVD) graphene sheet, and the fabrication diagram is displayed in Figure 2. We use a 475 µm-thick sapphire substrate with size of 1.5 cm × 1.5 cm. After ultrasonic cleaning with acetone and isopropanol separately, the sapphire substrate is spin-coated with a positive resist (polymethyl methacrylate) and baked at ~180 °C for 2 min. Subsequently, a 10-nm thick Al film is deposited with e-beam evaporator to make the substrate conductive, and the e-beam lithography process is used to fabricate a designed pattern on the sample with the dose of 1500 µC/cm^2^ and a beam resolution of 0.1 µm. After e-beam lithography, the sample has to be immersed in AZ351 (buffered NaOH and typically used in 1:4 dilution) for 10 min to remove Al firstly and developed in a solution made of Methyl Iso Butyl Ketone (MIBK):Isopropanol (IPA) = 1:3 for 1 min. The morphology of the gold metamaterials is depicted in Figure S1 (Supplementary Materials), which agrees well with the designed structure. The 5/50 nm-thick Ti/Au metal layer is then deposited on the surface with acetone applied to proceed the lift-off process. Subsequently, a graphene film is transferred to the metastructure/sapphire substrate by utilizing the wet etching process, following on deposition of electrode metal layer. The doping reagent for graphene is an ion-gel layer with high transmission in the mid-infrared region [39], which can be spin-coated on the surface of the sample. The ion-gel is prepared with 0.56 g of ionic liquid ([EMIM][TFSI]) dissolved in a mixture of 22 mg of PS-PEO-PS (10−44−10 kg mol^−1^) triblock copolymer and 20 mL dichloromethane. By using the mask with apertures, the electrode with proper scale can be obtained by engaging with the e-beam evaporation. Finally, wire-bonding technology is used to apply the electrical connection, and the scheme of the measured device is shown in Figure 3.

### 2.2. Terahertz Measurement and Characterization

An angular-solved THz-time domain spectroscopy (TDS) setup is used to measure the transmission properties at oblique incidence from the hybrid metal–graphene metastructure. The source laser is a Ti/Sapphire laser (Spectra Physics, MaiTai, CA, USA) with a pulse width of 200 fs and repetition rate of 75.9 MHz at 800 nm, which further impinge onto a photoconductive switch integrated on low-temperature grown GaAs (LT-GaAs) at an average power of 30 mW. The switch is biased at 40 V and produces the linear polarized THz pulses that propagate in a nitrogen-purged environment through the sample, with probe detection performed via an LT-GaAs-based photoconductive switch as well. The spot size of the THz pulse is approximately 3 mm with a frequency resolution of ~50 GHz. By rotating the sample, the incident angle of the light is typically set to 0–30° for the angular-resolved THz transmission measurement.

The morphology of the metastructure is characterized by field emission scanning electron microscopy (SEM, ZEISS GeminiSEM 300, ZEISS, Jena, Germany). Graphene properties are characterized by Raman spectroscopy (Laboratory Ram HR800, CA, USA, excitation wavelength at 532 nm). The electrical properties of the device are measured using a sourcemeter (Agilent 2400 and 2401).

### 2.3. Computational Method of Graphene

In calculations, the optical conductivity of graphene (*σ_g_*) can be described by altering the Fermi energy (*E_f_*) of graphene using the Kubo formula, which is present with both intraband and interband transitions [40,41]:

(1) $$\sigma_{g}=\sigma_{intra}+\sigma_{intra}$$

(2) $$\sigma_{intra}=-\frac{ie^{2}k_{b}T}{\pi \hbar^{2}(\omega +i2\Gamma )}[E_{f}/(k_{b}T)+2\ln(1+e^{\left[-E_{f}/(k_{b}T)\right]})]$$

(3) $$\sigma_{inter}=-\frac{ie^{2}}{4\pi \hbar }\ln(\frac{2E_{f}-(\omega +i4\pi \Gamma )\hbar }{2E_{f}+(\omega +i4\pi \Gamma )\hbar })$$

where *k_b_* and *T* is the Boltzmann constant and temperature, ℏ is reduced Plank constant, *Г* is the carrier relaxing time, *e* is the charge of an electron, and *ω* is angular frequency, respectively.

In the lower THz region, the optical conductivity of graphene is mainly governed by the intraband transition due to the Fermi level of graphene increasing above half of the photo energy. Here we only consider graphene with the $E_{f}\gg \hbar \omega$, hence the conductivity of graphene can be simplified to the Drude-like model [42]:

(4) $$\sigma_{g}(\omega )=\frac{e^{2}E_{f}}{\pi \hbar^{2}}\frac{i}{\omega +i\Gamma^{-1}}$$

where the carrier relaxing time *Г* defined by $\Gamma =\mu E_{f}/ev_{F}{}^{2}$ depends on the carriers mobility *μ* and the Fermi velocity $v_{F}$. Here, we employ *μ* of 10,000 cm^2^/(V•s) and $v_{F}$ of 1.1 × 10^6^ m/s throughout the entire calculations.

## 3. Results and Discussion

### 3.1. The Simulated Results of the Metal Metastructure

**Figure 4:**
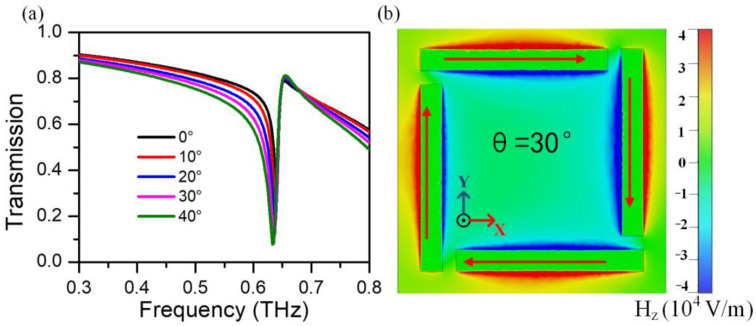
(**a**) Normalized transmission at oblique incidence under polarized wave along the x-direction. (**b**) Current flow (shown by arrow) and *z*-component of the magnetic field distribution at the resonance frequency (f = 0.64 THz) with an incident angle of 30°.

By using the frequency domain solver of CST Microwave Studio, the numerical calculations at oblique incidence can be performed with the unit cell boundary condition only supplying in *X*- and *Y*-directions. In simulations, the thickness of the metal and substrate are 50 nm and 2 µm, respectively. The permittivity of gold in the THz region is described by the Drude model with a plasma frequency of *ω_p_* = 1.37 × 10^16^ s^−1^ and damping constant *ω_d_* = 4.08 × 10^13^ s^−1^, respectively [43]. The dielectric constant of sapphire is approximately set as 11.7 [44]. The transmission spectra of the pure metamaterials are calculated, as shown in Figure 4a. It turns out that the transmission dip appears at around 0.64 THz at normal incidence, while the peak width broadens and shows slightly redshift with a rising incident angle. A snapshot of the current flow and the z-component of the magnetic field at the resonance frequency under oblique incidence is present in Figure 4b. Intuitively, the electric component of the incident light is paralleled to the plane of the metastructure under TE wave excitation, however, the magnetic field can couple to the capacitance of the split-rod metamaterials and induce a circular current in it, accounting for a magnetic dipole perpendicular to the surface plane of at ~0.64 THz. These metal split-rods provide inductances (L) and the split gaps serve a capacitance (C), which means the transmission dip (at 0.64 THz) is ascribed to the magnetic dipole mode. It is observed that the magnetic component of the incident light can induce a stronger circular current. This behavior is derived from the asymmetric incident electromagnetic fields on the plane of the split-rod metastructures, which can further produce phase delay between the adjacent unit cells along the x-direction. The electric component of the incident light interacts with the left arm of split-rod array firstly and induces charge accumulation at the left arm, which can counteract the external magnetic field. 

By investigating the electromagnetic response of the split-rod arrays, it is obvious that the gold split-rod metamaterials can be viewed as an LC resonance in which the effective inductance arises from the loop formed by the split-rod and effective capacitance due to the gap region between split-rod arms. The degree of asymmetry is increased by increasing the incident angle and further leads to the redshift of the resonance frequency. As a result, the electric field can effectively couple to the metastructures and induces a circular current, which accounts for a magnetic dipole perpendicular to the plane of metastructures.

### 3.2. The Electrical Response of the Hybrid Metal-Graphene Metastructure Device

**Figure 5:**
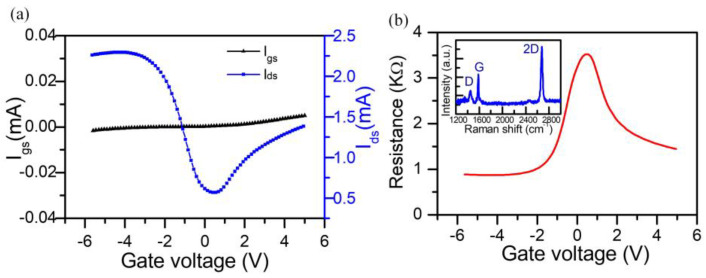
(**a**) The leakage current (*I_gs_*) and the source–drain current (*I_ds_*) of the hybrid metal–graphene metastructure device with different gate voltages. (**b**) The resistance of the measured device. The inset depicts the Raman spectrum of graphene film.

Due to the fact that carrier doping in graphene can be controlled with the gate voltage (*V_g_*), the electrical measurement is employed to study the chemical doping states of the device and helps to define the charge neutral point (CNP) of graphene, as given in Figure 5a. In the measurements, we use a source–drain voltage of *V_ds_* = 1.0 V. It is apparent that the leakage current (*I_gs_*) (black line) is stabilized around zero, while the source–drain current (*I_ds_*) (blue line) varies conspicuously with the applied gate voltage. This behavior reveals that the electric doping of graphene can be efficiently tunable, and the Fermi level of graphene can be adjusted approaching the Dirac point with a gate voltage of 0.5 V (i.e., *V_CNP_* = 0.5 V), which implies a natural p-type doping of our graphene samples. It is worth noting that the asymmetry response in the hole and electron transportation regions is observed, which is affected by current leakage through the electrical gating [45]. The calculated resistance of the graphene device is given in Figure 5b, which increases sharply from ~800 Ω to 3600 Ω with the gate voltage changing from −3 V to 0.5 V. This reveals that the electric doping of hybrid metal–graphene metastructure device can be efficiently regulated. Moreover, since the Fermi energy of graphene can be manipulated via an external voltage *V_g_*, the theoretical relation between *E_f_* and *V_g_* is given by [46]: $E_{f}=\hbar \nu_{F}\sqrt{\pi a\left|V_{g}-V_{CNP}\right|}$, where *a* = 1.45 × 10^13^/m^2^V is the capacitance per charge of the ion-gel [39]. According to this equation, the graphene Fermi energy could be effectively tuned from 0.05 to 0.3 eV by applying a top gate voltage from 0.5 V to −3 V. The inset in Figure 5b shows the Raman spectrum of graphene, indicating the film is identified as a monolayer. The thickness of graphene is measure via atomic force microscopy (AFM, Bruker, Dimension Icon, Bruker, Billerica, MA, USA), which confirms the monolayer structure in our experiment (Supplementary Materials).

### 3.3. The Modulation Performance of the Hybrid Metal-Graphene Metastructure Device

**Figure 6:**
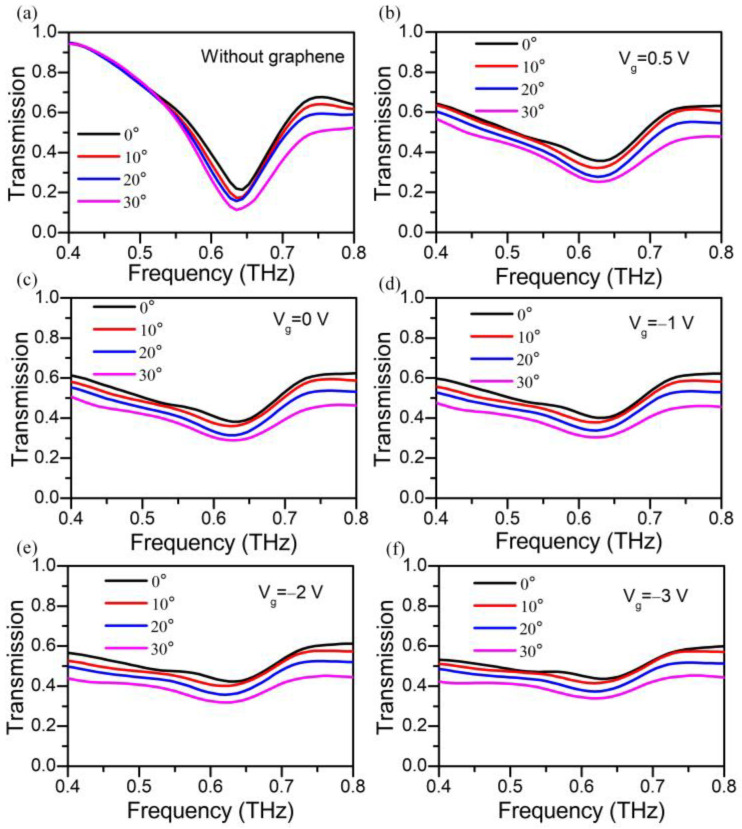
(**a**) Transmission spectra of the pure metastructure device at oblique incidence. (**b**) Transmission spectra of the gated metal-graphene metastructure device with different gate voltages of 0.5 V, (**c**) 0 V, (**d**) −1 V, (**e**) −2 V, and (**f**) −3 V, respectively. All spectra are normalized to the bare substrate.

The THz transmittance spectra of the pure metastructure fabricated on the sapphire substrate without graphene at different incidence after Fourier transformation of initial signal are presented in Figure 6a. The curves with different colors are measured with incident angle from 0° to 30°, and all spectra are normalized to the bare substrate. The result reveals a resonance peak at around 0.64 THz, and the absorption of bare metastructures enhances a little with the increasing incident angle, which fits the trend well with the simulation result (Figure 4a). This extremely sharp resonance of the sample without graphene layer indicates a strong LC resonance in the gold split-rod metamaterials. The THz transmittance spectra of the gated metal–graphene metastructure device at *V_CNP_ =* 0.5 V under different incidence are depicted in Figure 6b. When there is a graphene layer on the the surface of the metastructures, the resonance shrinks on a large scale but still shows a relatively deep and narrow resonance at ~0.62 THz. This is because the graphene behaves like a conducting layer in hybid metal–graphene metastructures. Once the graphene is placed under the surface of metastructure, it connects the ends of each split and enhances the losses in metastructures, which weakens the coupling effect between split-rods. The small but perceptible redshift of the LC resonance is due to the high permittivity of graphene associated with the relatively high conductivity arising from the sample fabrication and high carrier mobility. Meanwhile, the transmission of hybrid metal–graphene metastructure device decreases as the incident angle increases, which has a minimum value at the center of the resonance frequencies.

Figure 6c–f present the transmission of gated metal–graphene metastructure device with *V_g_* of 0 V, −1 V, −2 V and −3 V, which also indicate the resonance of hybrid metal–graphene metastructure device strongly rely on *V_g_*. The transmission of the device at the resonance frequency is promoted with increasing gate voltage and shows redshift at *θ* = 30°. By increasing the *V_g_* away from CNP, the highly doped graphene provides a direct conducting channel that shorts the charge oscillations across the slit, which significantly suppresses the local field enhancement. It can be seen that the existence of graphene strongly affects the optical response of the whole system, which contributes to the rising of the transmission, whereas the resonance peak still appears with voltage applied. It is worth noting that the transmission of the hybrid device cannot be tuned with further increases in the *V_g_*. This result is attributed to the doping saturation of ion-gel, which means the carriers concentration of graphene shows little difference when *V_g_* continues to increase.

**Figure 7:**
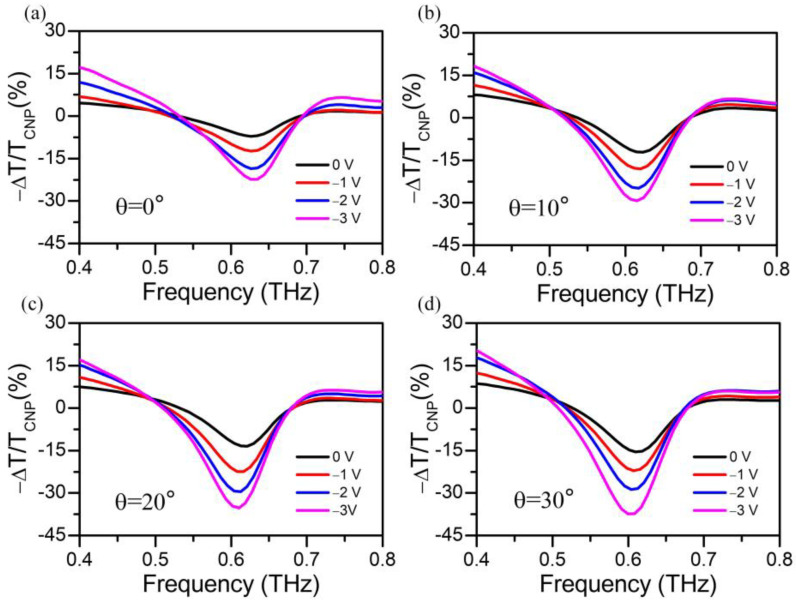
(**a**) Relative transmittance normalized value near the CNP for a selection of spectra (with *V_CNP_* = 0.5 V) with incident angle of 0°, (**b**) 10°, (**c**) 20° and (**d**) 30° with gate voltage of 0.5 V, 0 V, −1 V, −2 V, and −3 V, respectively.

In order to evaluate the modulation of the gated metal-graphene metastructure device more comprehensively, the modulation depth (MD, −Δ*T/T*_0_ *=* (*T_g_* − *T*_0_)/*T*_0_, *T_g_* and *T_0_* represent the transmission of the device with applied at *V_g_* and *V_CNP_*) is defined as the relative transmittance normalized to the value near the CNP for a selection of spectra for *V_CNP_* = 0.5 V under normal incidence, as shown in Figure 7a. Clearly, it can be derived that the modulation depth increases with a growing gate voltage, which can obtain −22.3% at 0.62 THz with *V_g_* = −3.0 V under normal incidence. The relative transmittance with incident angles of 10°, 20°and 30° are present in Figure 7b–d. With the rising of incident angle, the relative transmittance shows a similar trend as that at the normal incidence and can be further enhanced by the higher gating. The modulation depths at resonance frequencies center are calculated to be 28.9%, −34.5%, and −37.6% corresponding to incident angles of 10°, 20°and 30°, respectively. These results suggest an angle-dependent modulation of the hybrid metal–graphene metastructure device.

### 3.4. Angular-Dependent Modulation

**Figure 8:**
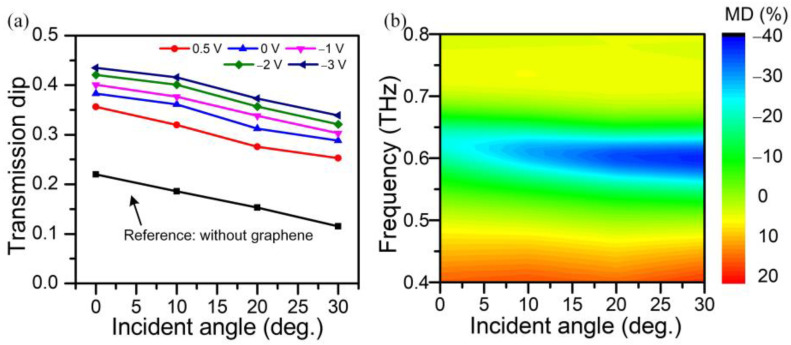
(**a**) Transmission dips with respect to the incident angle. (**b**) Relative transmittance normalized transmittance with *V_g_* = −3.0 V under oblique incidence.

To verify the angular-dependent modulation more intuitively, we investigate the transmission dip as a function of incident angle, as shown in Figure 8a. The transmission dip shows an approximately linear decay with the promoting incident angle, and the dropping trend of pure metastructures is similar to the hybrid metal–graphene metastructure device. By raising the incident angle, the transmission dip at resonant frequencies center can be derived as 0.354, 0.321, 0.276 and 0.251 with *V_g_* = −3.0 V corresponding to incident angles of 0°, 10°, 20°, and 30°, respectively. Moreover, the modulation depth of the hybrid metal–graphene metastructure device with *V_g_* = −3 V is depicted in Figure 8b, which demonstrates that the modulation depth around the resonance frequencies center can be promoted with the increasing angle. The angular-dependent modulation of hybrid metal–graphene metastructure device with smaller *V_g_* is given in Figure S3 (Supplementary materials), which shows a similar trend to that of *V_g_* = −3 V. It can be seen that the modulated characteristics show a strong dependency on the incident angle and the gate voltage. With an incident angle of 30°, the modulation depth can reach maximum (−37.6%) at around 0.62 THz with *V_g_* = −3.0 V.

### 3.5. Mechanism of Angular-Dependent Modulation

**Figure 9:**
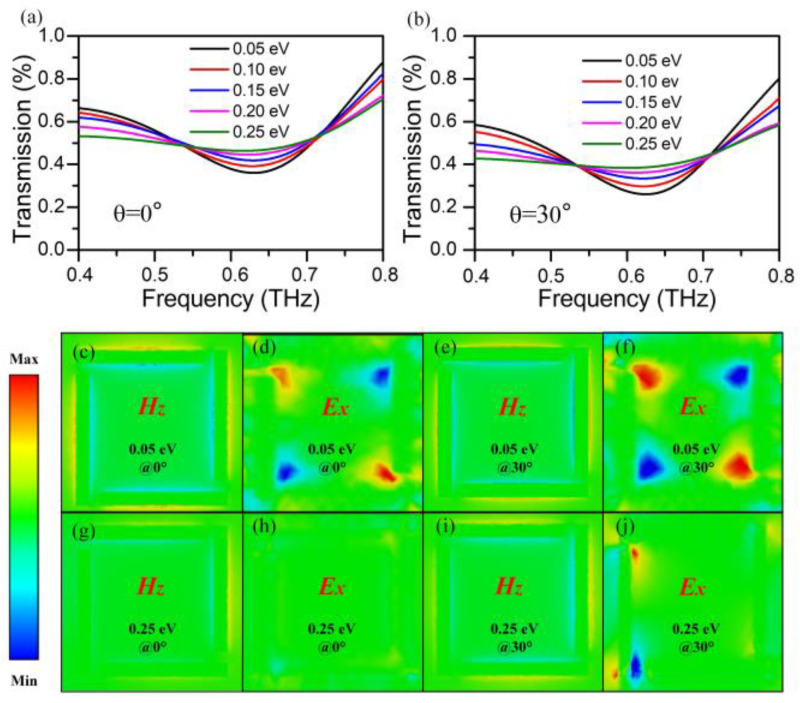
(**a**) Simulated THz transmission spectra with various Fermi energy of graphene probed with incident angle of 0° and (**b**) 30°. (**c**–**j**) The *z-*component distributions of the magnetic fields and *x-*component distributions of electric fields in the hybrid metal–graphene metastructure device by varying the incident angle for the LC resonance dip, respectively.

To gain a better understanding of the physical mechanisms of the angular-dependent modulation characteristics, the simulated transmission spectra of hybrid metal–graphene metastructure device are applied by numerical results versus the incident angle and the Fermi energy of graphene layer, as shown in Figure 9a,b. It is clear that the transmission dips are promoted with the enhancement of Fermi energy, but decrease with the increasing incident angle. These results are well-consistent with the spectra obtained in the experimental results in Figure 6. The slight resonance frequency difference for each result can be ascribed to the reason that the introduction of defect and quality reduction from graphene during the fabrication process is ignored, which is replaced by an ideal nanofilm close to surface of the metastructures in simulations. Due to screening the interaction between the incidence and artificial resonators, the near-field intensity for both magnetic and electric would be weakened.

Figure 9c,d show the *z*-component magnetic field and *x-*component electric field at frequencies centered within resonant modes under normal incidence, respectively. It is evident that the magnetic fields are mainly localized at the edge of individual split-rods with TE mode, which lead to a magnetic dipole perpendicular to the surface plane of metamaterials. Hence the circulating currents can be excited, which naturally induces opposite electrical fields in the *x*-direction. Similarly, the distribution of magnetic fields at oblique incidence shows further localization at the edge of individual split-rods as portrayed in Figure 9e, which indicate that the stronger resonant mode is produced by the asymmetric incident electromagnetic fields. Figure 9f depicts the induced circulating currents under oblique incidence is also in-phase, which is evidenced by the more destructive electric resonance modes. That is to say, the interaction between the graphene and metamaterials can be more strongly coupled at larger incident angle, which consequently makes the transmission dip much deeper. Indeed, this mode coupling with a substantially different phase variance is generally viewed as the LC effect by perturbating the symmetry by incidence-induced phase difference. Since the electrical control of modulated characteristics can be obtained by changing the Fermi energy of graphene, Figure 9g–j present the field distribution of the hybrid metal–graphene metastructure device under normal and oblique incidence when the Fermi level of graphene expands to 0.25 eV. It is worth noting that the near-field intensity for both magnetic and electric fields decrease with increasing Fermi energy, due to the high screening effect of graphene film causing the uncoupled interaction of LC resonance. Therefore, the angular-dependent modulation of hybrid graphene metastructure can be modified via both the incident angle and the gate voltage, which can make this device a reasonable application in narrow band switches/sensors and free us from the geometric restructure in most metamaterial-based devices.

## 4. Conclusions

In conclusion, in this work we investigate the angle-dependent modulation of electrostatic gating for a hybrid metal–graphene metastructure device. Through an experiment performed on an angle-resolved TDS setup, the maximum angular-dependent modulation depth is obtained as −37.6% with *V_g_* = −3 V at an incident angle of 30°. To further reveal the physical mechanism of the modulation properties, a theoretical analysis for transmitted dependency on the incident angle and Fermi energy of graphene is proposed. It is observed that the LC resonant model is employed to describe the near-field interaction in this hybrid device, which agrees well with the experimental results. The distribution of magnetic and electric fields further illuminates that the modulated behaviors lie in the recombination effect of the conductive graphene. These results prove that the angular-dependent modulation of the gated graphene-metastructure device can potentially provide a platform for strong light–matter interactions. Further tunable modulation of graphene-based metastrctures can be achieved by applying vertical or broadside coupling, which can be effective routes through exploiting the interlayer near-field electromagnetic coupling. Our works indicate that the graphene-based optical modulator can be used under multi-angle incident conditions.

## Data Availability

The data is available on reasonable request from the corresponding author.

## Supplementary Materials

The following supporting information can be downloaded at: https://www.mdpi.com/article/10.3390/nano13131914/s1, Figure S1: The SEM image of the gold metastructures, and the inset shows the higher-magnification image; Figure S2: AFM image of graphene. (b) The height profile of the film thickness along the red line; Figure S3: (a) Relative transmittance normalized transmittance with Vg = −1 V and (b) Vg = −2 V under oblique incidence, respectively.

## References

[1] Stantchev R.I., Sun B., Hornett S.M., Hobson P.A., Gibson G.M., Padgett M.J., Hendry E. (2016). Noninvasive, near-field terahertz imaging of hidden objects using a single-pixel detector. Sci. Adv..

[2] Koenig S., Lopez-Diaz D., Antes J., Boes F., Henneberger R., Leuther A., Tessmann A., Schmogrow R., Hillerkuss D., Palmer R. (2013). Wireless sub-THz communication system with high data rate. Nat. Photonics.

[3] Tan C., Wang S., Li S., Liu X., Wei J., Zhang G., Ye H. (2022). Cancer Diagnosis Using Terahertz-Graphene-Metasurface-Based Biosensor with Dual-Resonance Response. Nanomaterials.

[4] Zhang X.C., Shkurinov A., Zhang Y. (2017). Extreme terahertz science. Nat. Photonics.

[5] Kono S., Tani M., Sakai K. (2001). Ultrabroadband photoconductive detection: Comparison with free-space electro-optic sampling. Appl. Phys. Lett..

[6] Libon I.H., Baumgärtner S., Hempel M., Hecker N.E., Feldmann J., Koch M., Dawson P. (2000). An optically controllable terahertz filter. Appl. Phys. Lett..

[7] Yoo H.K., Yoon Y., Lee K., Kang C., Kee C., Hwang I., Lee J.W. (2014). Highly efficient terahertz wave modulators by photo-excitation of organics/silicon bilayers. Appl. Phys. Lett..

[8] Kowerdziej R., Olifierczuk M., Parka J. (2018). Thermally induced tunability of a terahertz metamaterial by using a specially designed nematic liquid crystal mixture. Opt. Express.

[9] Zaman A.M., Lu Y., Romain X., Almond N.W., Burton O.J., Alexander-Webber J., Hofmann S., Mitchell T., Griffiths J.D.P., Beere H.E. (2022). Terahertz metamaterial optoelectronic modulators with GHz reconfiguration speed. IEEE T. THZ Sci. Techn..

[10] Castro Neto A.H., Guinea F., Peres N.M.R., Novoselov K.S., Geim A.K. (2009). The electronic properties of graphene. Rev. Mod. Phys..

[11] Li G., Fu J., Sun F., Nie C., Wu J. (2023). Graphene/Ge Photoconductive Position-Sensitive Detectors Based on the Charge Injection Effect. Nanomaterials.

[12] Bonaccorso F., Sun Z., Hasan T., Ferrari A. (2010). Graphene photonics and optoelectronics. Nat. Photonics.

[13] Bao Q., Loh K.P. (2012). Graphene photonics, plasmonics, and broadband optoelectronic devices. ACS Nano.

[14] Zhu Y., Murali S., Cai W., Li X., Suk J.W., Potts J.R., Ruoff R.S. (2010). Graphene and graphene oxide: Synthesis, properties, and applications. Adv. Mater..

[15] Mak K.F., Ju L., Wang F., Heinz T.F. (2012). Optical spectroscopy of graphene: From the far infrared to the ultraviolet. Solid State Commun..

[16] Wang L., An N., He X., Zhang X., Zhu A., Yao B., Zhang Y. (2022). Dynamic and active THz graphene metamaterial devices. Nanomaterials.

[17] Sensale-Rodriguez B., Fang T., Yan R., Kelly M.M., Jena D., Liu L., Xing H. (2011). Unique prospects for graphene-based terahertz modulators. Appl. Phys. Lett..

[18] Lv J., Zhou M., Gu Q., Jiang X., Ying Y., Si G. (2019). Metamaterial lensing devices. Molecules.

[19] Martinez F., Maldovan M. (2022). Metamaterials: Optical, acoustic, elastic, heat, mass, electric, magnetic, and hydrodynamic cloaking. Mater. Today Phys..

[20] Cen C., Chen Z., Xu D., Jiang L., Chen X., Yi Z., Wu P., Li G., Yi Y. (2020). High quality factor, high sensitivity metamaterial graphene—Perfect absorber based on critical coupling theory and impedance matching. Nanomaterials.

[21] Lochbaum A., Dorodnyy A., Koch U., Koepfli S.M., Volk S., Fedoryshyn Y., Wood V., Leuthold J. (2020). Compact mid-infrared gas sensing enabled by an all-metamaterial design. Nano Lett..

[22] Shen S., Liu X., Shen Y., Qu J., Pickwell-MacPherson E., Wei X., Sun Y. (2022). Recent advances in the development of materials for terahertz metamaterial sensing. Adv. Opt. Mater..

[23] Buchnev O., Podoliak N., Kaltenecker K., Walther M., Fedotov V.A. (2020). Metasurface-based optical liquid crystal cell as an ultrathin spatial phase modulator for THz applications. ACS Photonics.

[24] Hemmati H., Bootpakdeetam P., Magnusson R. (2019). Metamaterial polarizer providing principally unlimited extinction. Opt. Lett..

[25] Yang J., Chen J., Quan L., Zhao Z., Shi H., Liu Y. (2021). Metamaterial-inspired optically transparent active dual-band frequency selective surface with independent wideband tunability. Opt. Express.

[26] Jahani S., Jacob Z. (2016). All-dielectric metamaterials. Nat. Nanotechnol.

[27] Choi M., Lee S.H., Kim Y., Kang S.B., Shin J., Kwak M.H., Kang K.Y., Lee Y.H., Park N., Min B. (2011). A terahertz metamaterial with unnaturally high refractive index. Nature.

[28] Liu D.-J., Xiao Z.-Y., Ma X.-L., Wang Z.-H. (2015). Asymmetric transmission of linearly and circularly polarized waves in metamaterial due to symmetry-breaking. Appl. Phys. Express.

[29] Li Y., Xu Y., Jiang J., Cheng S., Yi Z., Xiao G., Zhou X., Wang Z., Chen Z. (2023). Polarization-sensitive multi-frequency switches and high-performance slow light based on quadruple plasmon-induced transparency in a patterned graphene-based terahertz metamaterial. Phys. Chem. Chem. Phys..

[30] Valmorra F., Scalari G., Maissen C., Fu W., Schönenberger C., Choi J.W., Park H.G., Beck M., Faist J. (2013). Low-bias active control of terahertz waves by coupling large-area CVD graphene to a terahertz metamaterial. Nano Lett..

[31] Jessop D.S., Kindness S.J., Xiao L., Braeuninger-Weimer P., Lin H., Ren Y., Ren C.X., Hofmann S., Zeitler J.A., Beere H.E. (2016). Graphene based plasmonic terahertz amplitude modulator operating above 100 MHz. Appl. Phys. Lett..

[32] Shi S.F., Zeng B., Han H.L., Hong X., Tsai H.Z., Jung H.S., Zettl A., Crommie M.F., Wang F. (2015). Optimizing broadband terahertz modulation with hybrid graphene/metasurface structures. Nano Lett..

[33] Balci O., Kakenov N., Karademir E., Balci S., Cakmakyapan S., Polat E.O., Caglayan H., Özbay E., Kocabas C. (2018). Electrically switchable metadevices via graphene. Sci. Adv..

[34] Zhou Y., Yiwen E., Xu X., Li W., Wang H., Zhu L., Bai J., Ren Z., Wang L. (2016). Angular dependent anisotropic terahertz response of vertically aligned multi-walled carbon nanotube arrays with spatial dispersion. Sci. Rep..

[35] Sun G., Peng S., Zhang X., Zhu Y. (2020). Switchable electromagnetically induced transparency with toroidal mode in a graphene-loaded all-dielectric metasurface. Nanomaterials.

[36] Zhang Y., Wang L., Zhang Z. (2017). Circular dichroism in planar achiral plasmonic L-shaped nanostructure arrays. IEEE Photonics J..

[37] Nguyen T.T., Lim S. (2017). Bandwidth-enhanced and wide-angle-of-incidence metamaterial absorber using a hybrid unit cell. Sci. Rep..

[38] Gao K., Cao X., Gao J., Li T., Yang H., Li S. (2022). Ultrawideband metamaterial absorber for oblique incidence using characteristic mode analysis. Photonics Res..

[39] Fang Z., Wang Y., Schlather A.E., Liu Z., Ajayan P.M., de Abajo F.J., Nordlander P., Zhu X., Halas N.J. (2014). Active tunable absorption enhancement with graphene nanodisk arrays. Nano Lett..

[40] Majidi M.A., Siregar S., Rusydi A. (2015). Theoretical study of optical conductivity of graphene with magnetic and nonmagnetic adatoms. Phys. Rev. B.

[41] Low T., Avouris P. (2014). Graphene plasmonics for terahertz to mid-infrared applications. ACS Nano.

[42] Koulouklidis A.D., Tasolamprou A.C., Doukas S., Kyriakou E., Ergoktas M.S., Daskalaki C., Economou E.N., Kocabas C., Lidorikis E., Kafesaki M. (2022). Ultrafast terahertz self-induced absorption and phase modulation on a graphene-based thin film absorber. ACS Photonics.

[43] Georgiev P., Simeonova S., Tsekov R., Balashev K. (2020). Dependence of plasmon spectra of small gold nanoparticles from their size: An atomic force microscopy experimental approach. Plasmonics.

[44] Gu J., Singh R., Liu X., Zhang X., Ma Y., Zhang S., Maier S.A., Tian Z., Azad A.K., Chen H.T. (2012). Active control of electromagnetically induced transparency analogue in terahertz metamaterials. Nat. Commun..

[45] Ye X., Du Y., Wang M., Liu B., Liu J., Jafri S.H.M., Liu W., Papadakis R., Zheng X., Li H. (2023). Advances in the field of two-dimensional crystal-based photodetectors. Nanomaterials.

[46] Liu C., Liu P., Yang C., Lin Y., Liu H. (2019). Analogue of dual-controlled electromagnetically induced transparency based on a graphene met amaterial. Carbon.

