# Supplementary material for: Angular-Dependent THz Modulator with Hybrid Metal-Graphene Metastructures
Source: Nanomaterials (Basel). 2023 Jun 23;13(13):1914. doi: 10.3390/nano13131914 (PMC10343880; doi:10.3390/nano13131914)
Supplement: Supplementary file 1 [file nanomaterials-13-01914-s001.zip › nanomaterials-2434754-supplementary.pdf]

nanomaterials

Angular-Dependent THz Modulator with Hybrid Metal-Graphene Metastructures

Huan Wang [1,*], Jiajun Linghu [1], Xuezhi Wang [1], Qiyi Zhao [2] and Hao Shen [1,*]

[1]School of Science, Chang'an University, Xi'an, 710061, China.
[2]School of Science, Xi'an University of Posts & Telecommunications, Xi'an 710121, China
* Correspondence: tcoswh@chd.edu.cn; haoshen@chd.edu.cn

The morphology of the metal metastructure

Figure S1 shows an SEM image of the gold metastructures, and the inset shows a higher-magnification image, which was measured via scanning electron microscopy (SEM, FEI Quanta 400 ESEM-FEG). This result verifies that the fabricated metastructures agree well with the designed structure.

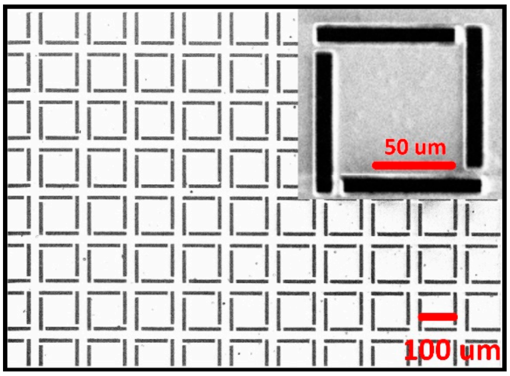

Figure S1. The SEM image of the gold metastructures, and the inset shows the higher-magnification image.

The thickness of as-deposited graphene

Atomic force microscopy (AFM, Bruker, Dimension Icon) is carried out to determine the thickness of the CVD grapheme, as shown in Figure S2. The results demonstrate that the thickness of graphene is approximately 0.72 nm. Due to the existence of wrinkles in the AFM image, the CVD grapheme in our experiment should be monolayer, which is consistent with the Raman result (shown in Figure 5).

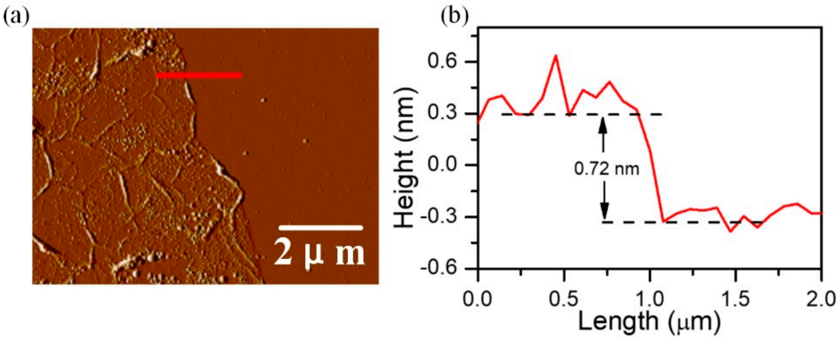

Figure S2. AFM image of graphene. (b) The height profile of the film thickness along the red line.

Angular-dependent modulation of hybrid metal–graphene metastructure device with different gate voltage

The modulation depths of the hybrid metal–graphene metastructure device with Vg=-1 V and Vg=-2 V are depicted in Figure S3a and S3b, respectively. It is apparent that the modulated characteristics show a strong dependency on the incident angle and the gate voltage, which presents a similar trend with Figure 7b. Notably, the modulation depth shows smaller values with the V_g=-1 V compared with the higher gate voltage.

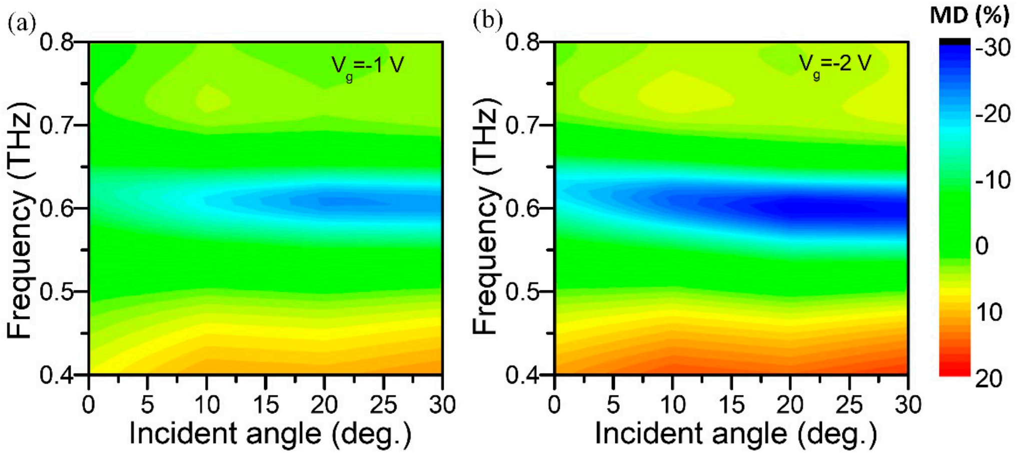

Figure S3. (a) Relative transmittance normalized transmittance with V_g=−1 V and (b) V_g=−2 V under oblique incidence, respectively.
